# Survival Impact of Cytoreductive Surgery in FIGO Stage IVB Endometrial Cancer: A Population-Based Study

**DOI:** 10.3390/cancers17243965

**Published:** 2025-12-12

**Authors:** Paolo Gennari, Andrea Willeke, Atanas Ignatov

**Affiliations:** Department of Gynecology and Obstetrics, Otto-Von-Guericke University, 39108 Magdeburg, Germany

**Keywords:** endometrial cancer, cytoreductive surgical procedures, metastatic disease

## Abstract

**Background:** The impact of cytoreductive surgery on survival in patients with FIGO stage IVB endometrial cancer remains controversial. This study aimed to evaluate the independent effect of surgery within contemporary multimodal treatment strategies using a large population-based registry. **Patients and methods:** We conducted a multicenter retrospective analysis of 6582 patients with endometrial cancer recorded in the Saxony-Anhalt cancer registry between 2000 and 2020. After exclusions, 294 patients with FIGO stage IVB disease were included. Disease-free survival (DFS) was the primary outcome and overall survival (OS) the secondary outcome. Patients were initially categorized into three treatment groups: surgery only, non-surgical therapy, and combined multimodal therapy. To address potential selection bias, a propensity score-based inverse probability of treatment weighting (IPTW) analysis was performed to compare surgery versus no surgery. Time-period subgroup analysis was conducted for 2000–2010 and 2011–2019. **Results:** The median follow-up was 68 months (range 0–265 months). Combination therapy resulted in significantly improved median DFS and OS compared to surgery or non-surgical therapy alone. After IPTW adjustment, surgery was independently associated with improved OS (HR 0.64, 95% CI 0.53–0.78, *p* < 0.01) and DFS (HR 0.67, 95% CI 0.48–0.94, *p* = 0.02). Subgroup analysis demonstrated a significant survival benefit of surgery in the contemporary period (2011–2019), whereas the effect was weaker and not statistically significant in the earlier period (2000–2010). **Conclusions:** Cytoreductive surgery is independently associated with improved survival in patients with FIGO stage IVB endometrial cancer, particularly within modern therapeutic eras. These findings support the integration of surgery as part of a multimodal approach in selected patients while highlighting the importance of refined patient selection in the context of evolving systemic therapies.

## Simple Summary

Patients with FIGO stage IVB endometrial cancer have limited treatment options, and the role of cytoreductive surgery remains uncertain. Using a large population-based registry, we evaluated whether surgery provides a survival advantage compared with non-surgical treatment in real-world clinical practice. After adjusting for differences between patients through inverse probability of treatment weighting, cytoreductive surgery was associated with significantly improved overall and disease-free survival. The benefit of surgery was particularly evident in patients treated in the modern therapeutic era (2011–2019). These findings support the role of surgery as part of a multimodal treatment approach in selected patients with metastatic endometrial cancer.

## 1. Introduction

Endometrial cancer is the most common gynecologic malignancy in high-income countries, and its incidence continues to rise worldwide. While the majority of patients are diagnosed at an early stage and experience favorable outcomes, approximately 10–15% present with advanced or metastatic disease at primary diagnosis, corresponding to FIGO stage IVB. This subgroup represents a biologically heterogeneous and clinically challenging population characterised by limited long-term survival and the absence of a universally accepted standard of care, particularly regarding the role of surgery in the presence of distant metastases [1,2].

In recent years, the understanding and management of endometrial cancer have undergone a paradigm shift driven by advances in molecular classification and the development of targeted therapies. The Cancer Genome Atlas (TCGA) has enabled stratification of endometrial cancer into four molecular prognostic categories: POLE-ultramutated, microsatellite instability-high (MMR-deficient), copy-number low, and copy-number high/p53-abnormal tumors [3,4,5]. This molecular framework has been incorporated into ESGO/ESTRO/ESP and NCCN guidelines, influencing both prognostication and treatment selection. In parallel, immune checkpoint inhibitors (ICIs), such as pembrolizumab and dostarlimab, have demonstrated significant clinical benefit in advanced and recurrent MMR-deficient endometrial cancer, while combination regimens involving ICIs and chemotherapy have expanded therapeutic options even for MMR-proficient tumors [6,7,8]. Moreover, emerging data on PARP inhibitors, HER2-targeted therapies, and antibody–drug conjugates further underscore the rapidly evolving systemic treatment landscape for advanced disease.

Despite these advances, the optimal role of surgery in FIGO stage IVB endometrial cancer remains controversial. Traditionally, the presence of distant metastases has been regarded as a relative contraindication to extensive surgical intervention, with systemic therapy favored as the primary treatment modality. However, growing evidence suggests that cytoreductive surgery may confer a survival benefit in carefully selected patients, particularly when complete macroscopic resection can be achieved [9,10,11]. Several retrospective studies have reported improved overall survival in patients undergoing aggressive cytoreduction in combination with systemic therapy compared to non-surgical treatment alone; nevertheless, these findings are limited by small sample sizes, inherent selection bias, and considerable heterogeneity in treatment strategies.

Importantly, most available studies predate the widespread implementation of molecular profiling and modern systemic therapies, leaving a critical gap in understanding how cytoreductive surgery fits into contemporary multimodal treatment strategies [12]. Furthermore, real-world population-based evidence exploring treatment patterns and clinical outcomes in this high-risk subgroup remains scarce.

Therefore, the aim of the present study was to evaluate the survival impact of cytoreductive surgery in patients with FIGO stage IVB endometrial cancer using a large population-based tumor registry. Specifically, we compared disease-free survival and overall survival among patients treated with surgery alone, non-surgical therapy alone, and combined multimodal treatment. By analyzing real-world data over an extended period, this study seeks to contribute to the ongoing debate concerning the role of surgery in metastatic endometrial cancer and to determine whether cytoreductive approaches are associated with improved outcomes in routine clinical practice.

## 2. Materials and Methods

### 2.1. Patients

We investigated cases of endometrial cancer diagnosed between January 2000 and December 2020 that were recorded in the prospectively maintained regional cancer registry of Saxony-Anhalt, a federal state of Germany. This population-based tumor registry contains prospectively collected data on patient demographics, diagnosis, age, tumor stage, tumor grading, lymph node status, date of diagnosis, date and site of recurrence, date of death, and treatment modalities used [13]. The registry covers a population of approximately 1.2 million individuals in northern Saxony-Anhalt. Information regarding the date and cause of death is automatically updated by the main health authority shortly after death.

**Figure 1:**
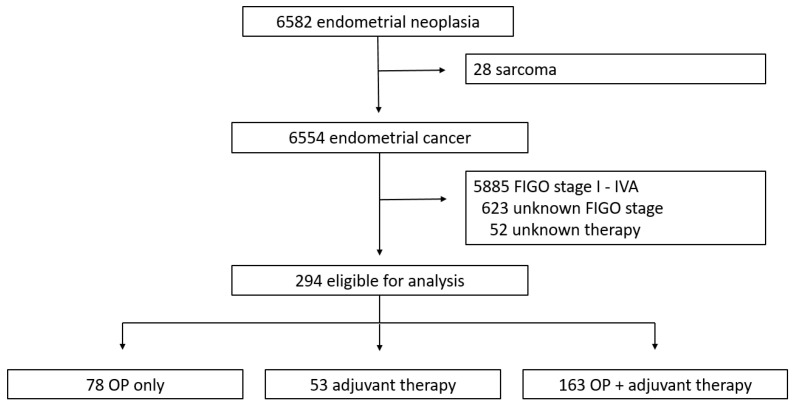
Study flow diagram.

From this cohort, we selected all patients diagnosed with FIGO stage IVB endometrial cancer (Figure 1). During the study period, 6582 patients with endometrial malignancies were identified, of whom 6554 had histologically confirmed endometrial cancer (Figure 1). Patients with FIGO stages other than IVB (*n* = 5885), unknown FIGO stage (*n* = 623), or unknown treatment status (*n* = 52) were excluded. The final study population comprised 294 patients, who were categorized into three groups according to treatment modality: surgery only (OP), non-surgical treatment consisting of radiotherapy and/or chemotherapy and/or hormonal therapy (RT/CT/HT), and combined treatment including surgery plus radiotherapy and/or chemotherapy and/or hormonal therapy (OP + RT/CT/HT).

A substantial number of patients were excluded due to missing FIGO staging or incomplete documentation of treatment modalities, reflecting inherent limitations of population-based registry data, particularly in the earlier years of the study period. These exclusions were necessary to ensure accurate classification and reliable comparison between treatment groups.

Patients were considered for surgical treatment following multidisciplinary evaluation and based on predefined clinical criteria. Surgery was primarily offered to patients with acceptable performance status (ECOG 0–2), limited tumor burden, and the potential to achieve at least partial macroscopic tumor reduction. Additional determinants included the distribution of metastatic disease, radiological resectability, absence of rapidly progressive disease, and anticipated perioperative risk. Conversely, patients with poor performance status, extensive unresectable metastases, or high surgical risk were preferentially managed with non-surgical approaches.

These criteria reflect routine clinical decision-making in advanced endometrial cancer and guided the allocation of patients to surgical or non-surgical treatment strategies throughout the study period.

For descriptive purposes, non-surgical treatments comprised systemic and/or locoregional therapies, including chemotherapy, radiotherapy, hormonal therapy, and immunotherapy, administered either alone or in combination. The distribution of non-surgical modalities was heterogeneous: chemotherapy and radiotherapy were each administered to approximately 130 patients, whereas immunotherapy was used in 43 patients, predominantly in more recent years, reflecting the historical nature of a large proportion of the cohort. Moreover, considerable overlap between treatment modalities was observed, as many patients received combined systemic and locoregional approaches. Although chemotherapy and radiotherapy were frequently used, further stratification according to specific non-surgical modality (e.g., chemotherapy-based, radiotherapy-based, or immunotherapy-based regimens) would have resulted in multiple small and clinically heterogeneous subgroups, thereby reducing statistical power and limiting interpretability, particularly given the relatively low number of patients receiving immunotherapy. Therefore, non-surgical treatments were analysed as a single comparator group. This strategy was chosen to avoid over-fragmentation of the cohort and minimize the risk of unstable estimates, while the primary focus of the study remained the evaluation of the independent impact of surgery on survival outcomes. This limitation restricts direct comparability between specific non-surgical strategies and should be considered when interpreting the results.

The primary outcome measure was disease-free survival (DFS), defined as the interval from the date of diagnosis to local and/or regional recurrence, distant metastasis, or death from disease, whichever occurred first. Patients who died from other causes or with an unknown cause of death were censored. Follow-up ended at the time of death, the date of last available information, or the last registry update on 31 December 2019.

The manuscript was prepared in accordance with the Strengthening the Reporting of Observational Studies in Epidemiology (STROBE) guidelines [14].

Ethical approval was granted by the Research and Ethics Committee of Otto-von-Guericke University, Magdeburg, Germany. All procedures were conducted in accordance with the Declaration of Helsinki and Good Clinical Practice guidelines. According to the statement of the Ethics Committee, individual informed consent was not required because the study used anonymized registry data.

### 2.2. Statistical Analysis

The statistical analyses were performed using SPSS software, version 29.0 (IBM SPSS Statistics, Chicago, IL, USA). Clinical, pathological, and treatment-related variables were compared between groups using the chi-squared test or Fisher’s exact test for categorical variables, and the two-sample *t*-test for continuous variables (age). Survival probabilities were estimated using the Kaplan–Meier method, and differences between survival curves were assessed using the log-rank test. All statistical tests were two-sided, and a *p*-value < 0.05 was considered statistically significant.

#### Propensity Score and IPTW Analysis

To address potential selection bias resulting from the non-random allocation of surgical treatment, a propensity score-based inverse probability of treatment weighting (IPTW) approach was applied. For this purpose, patients were dichotomized into two groups: (1) a surgery group, including patients who underwent cytoreductive surgery with or without adjuvant radiotherapy and/or chemotherapy, and (2) a no-surgery group, consisting of patients treated with radiotherapy and/or chemotherapy alone. This classification was selected to evaluate the independent impact of surgery on survival outcomes.

The propensity score for undergoing surgery was estimated using multivariable logistic regression, incorporating the following baseline covariates: age at diagnosis, histological subtype (endometrioid vs. non-endometrioid), tumor grade, number of metastatic sites, and distribution of metastases (bone, lung, liver, brain, lymph nodes, and abdominal/peritoneal metastases). Stabilized IPTW weights were calculated as the inverse of the probability of having received the treatment actually administered and were applied to all subsequent survival analyses.

Covariate balance before and after weighting was assessed using standardized mean differences (SMD), with an absolute SMD < 0.10 considered indicative of adequate balance. Balance between groups was visualized using Love plots.

Overall survival (OS) was defined as the interval from the date of diagnosis to death from any cause or last follow-up. Disease-free survival (DFS) was defined as the time from diagnosis to first recurrence or death, whichever occurred first. IPTW-weighted Kaplan–Meier curves were generated for both OS and DFS, and IPTW-weighted Cox proportional hazards models with robust variance estimation were used to calculate hazard ratios (HRs) and corresponding 95% confidence intervals (CIs). All IPTW analyses were performed using Python version 3.12.

In addition, a time-period subgroup analysis was conducted to evaluate whether the effect of surgery on survival outcomes varied across different therapeutic eras. Patients were stratified according to the year of diagnosis into two periods: 2000–2010 and 2011–2019. Within each period, separate IPTW-weighted Cox proportional hazards models were applied for OS and DFS, comparing surgery versus no surgery. This analysis was performed to assess whether the magnitude and statistical significance of the surgical effect remained consistent over time.

## 3. Results

The median follow-up was 68 months (range 0–265 months). In total, 294 patients with FIGO stage IVB endometrial cancer and known treatment modalities were included in the final analysis. Baseline clinical and pathological characteristics are summarized in Table 1. Variables analyzed included patient age, histological subtype, tumor grade, and the pattern of distant metastases at diagnosis.

A significant difference in age distribution was observed among treatment groups (*p* < 0.001). The median age was 71 years (range 42–91) in the OP group, 72 years (range 40–85) in the RT/CT/HT group, and significantly lower in the OP + RT/CT/HT group at 65 years (range 28–84). Endometrioid histology accounted for 50.6% of tumors in the OP group, 65.5% in the RT/CT/HT group, and 58.2% in the OP + RT/CT/HT group, whereas non-endometrioid histology was observed in 45.7%, 32.7%, and 37.3%, respectively. Other histological subtypes were rare across all groups. These differences were not statistically significant (*p* = 0.458).

Tumor grade distribution was also comparable, with no significant differences between groups (*p* = 0.425). High-grade tumors predominated in all cohorts, accounting for 66.7% in the OP group, 55.6% in the RT/CT/HT group, and 62.6% in the combination therapy group.

Regarding metastatic patterns, abdominal and pulmonary sites were the most commonly affected at diagnosis (Table 1). Bone metastases were significantly more frequent in the RT/CT/HT group (21.8%) compared with the OP group (2.5%) and the OP + RT/CT/HT group (6.3%) (*p* < 0.001). Lung metastases were also most prevalent in the RT/CT/HT group (50.9%). In addition, abdominal/peritoneal metastases differed significantly between groups, occurring in 59.3% of the OP group, 27.3% of the RT/CT/HT group, and 60.8% of the OP + RT/CT/HT group (*p* < 0.001). Metastases to the liver, brain, distant lymph nodes and other sites were similarly distributed across groups.

### 3.1. Survival Analysis

**Figure 2:**
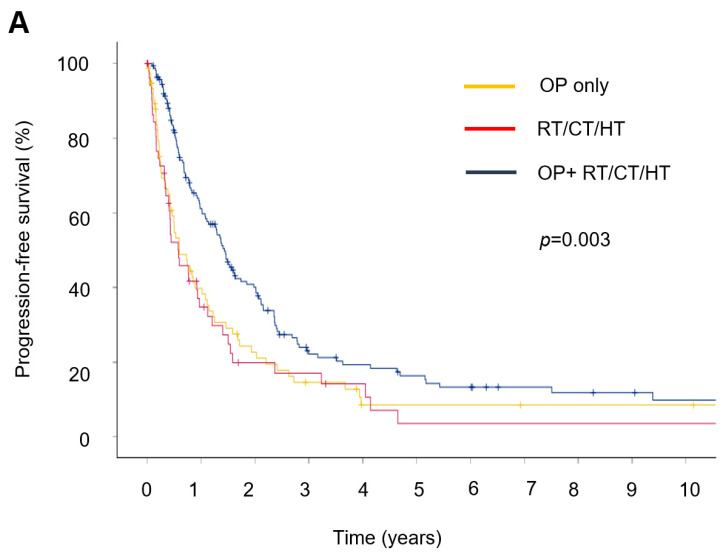

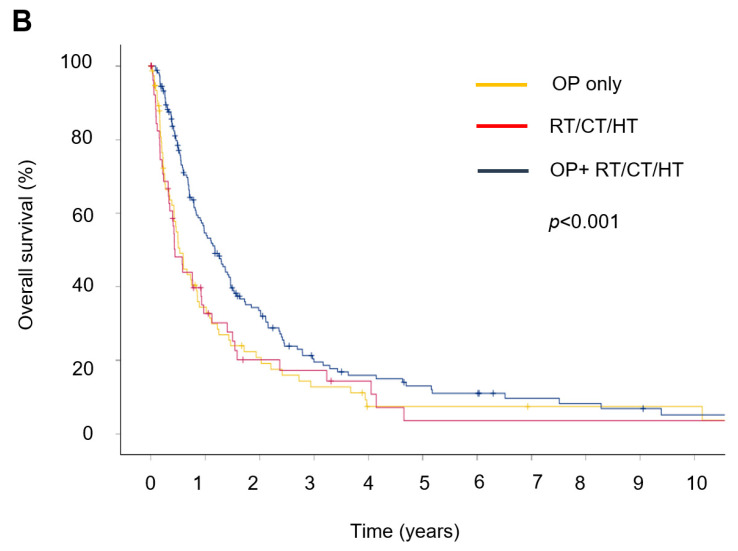
Kaplan–Meier survival curves for patients with FIGO stage IVB endometrial cancer stratified by treatment modality. (**A**) Progression-free survival (PFS). Combination therapy (surgery + radiotherapy and/or chemotherapy) achieved significantly longer PFS compared with surgery alone or non-surgical treatment alone. (**B**) Overall survival (OS). Combination therapy resulted in significantly improved OS compared with surgery alone and non-surgical treatment alone.

Subsequently, the survival impact of treatment was evaluated. The median progression-free survival (PFS) was significantly longer in patients treated with OP + RT/CT/HT (14.7 months) compared with those treated with surgery alone (7.1 months) or RT/CT/HT alone (6.9 months) (Figure 2A). Similarly, median overall survival (OS) was significantly prolonged in the combination therapy group (17.2 months) compared with the OP group (9.2 months) and the RT/CT/HT group (7.1 months) (Figure 2B).

Univariable and multivariable Cox regression analyses were performed to assess factors associated with PFS (Table 2). Compared with combination therapy, RT/CT/HT alone was associated with significantly worse PFS (HR = 0.62, 95% CI: 0.44–0.88, *p* = 0.007 in univariable analysis; HR = 0.58, 95% CI: 0.39–0.86, *p* = 0.007 in multivariable analysis). No significant difference in PFS was observed between RT/CT/HT and surgery alone. Age > 69 years was associated with worse PFS in univariable analysis but lost significance after adjustment. High tumor grade remained a strong independent predictor of poorer PFS in both analyses. Non-endometrioid histology was associated with worse PFS in univariable analysis but not after multivariable adjustment. Patients with more than two metastatic sites had significantly worse PFS compared with those presenting with a single metastasis, an association that remained significant after adjustment. The presence of bone or visceral metastases did not significantly influence PFS.

For OS (Table 3), combination therapy demonstrated a clear survival advantage compared with RT/CT/HT alone (HR = 0.55, 95% CI: 0.39–0.78, *p* < 0.001 in univariable analysis; HR = 0.49, 95% CI: 0.33–0.73, *p* < 0.001 in multivariable analysis). No significant difference in OS was found between RT/CT/HT and surgery alone. Age > 69 years, high tumor grade, and non-endometrioid histology were associated with worse OS, while the number of metastatic sites showed a borderline effect in the multivariable model. Bone and visceral metastases were not independently associated with OS.

### 3.2. IPTW-Adjusted Survival Analysis

**Figure 3:**
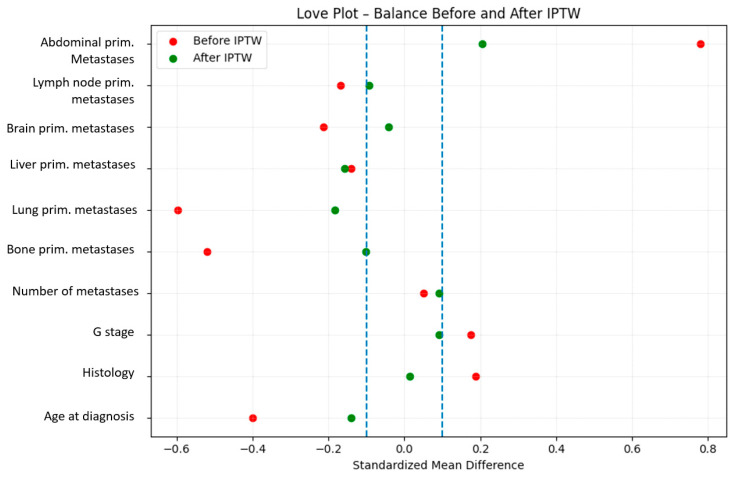
Covariate balance after inverse probability of treatment weighting. Love plot showing standardized mean differences (SMD) for baseline covariates before and after IPTW. After weighting, all covariates achieved adequate balance, with most SMD values < 0.10, indicating effective reduction in baseline differences between the surgical and non-surgical groups. The same IPTW model was applied for both overall survival and disease-free survival analyses.

**Figure 4:**
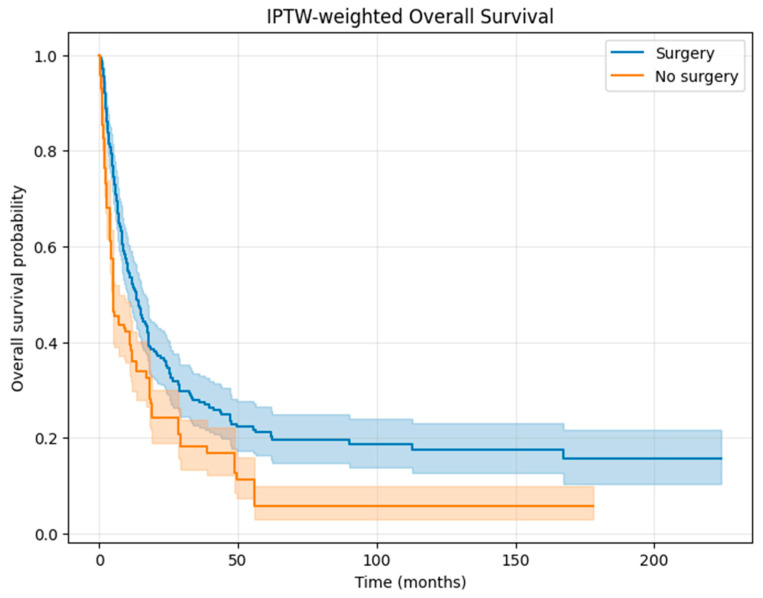
IPTW-weighted overall survival. Kaplan–Meier curves for overall survival (OS) in patients with FIGO stage IVB endometrial cancer treated with surgery versus non-surgical management, after adjustment using inverse probability of treatment weighting (IPTW). Shaded areas represent 95% confidence intervals.

After IPTW adjustment, baseline covariates between the surgery and no-surgery groups were well balanced, with all standardized mean differences below 0.10 (Figure 3, Love plot). IPTW-weighted Kaplan–Meier analysis demonstrated significantly improved OS in patients undergoing surgery compared with those receiving non-surgical therapy alone (Figure 4). In the IPTW-weighted Cox regression model, surgery was associated with a significantly reduced risk of death (HR 0.64, 95% CI 0.53–0.78, *p* < 0.01).

**Figure 5:**
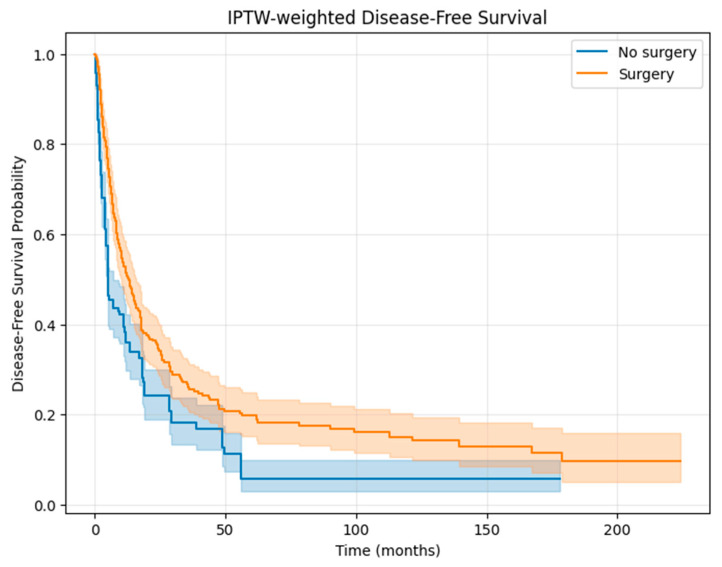
IPTW-weighted disease-free survival. Kaplan–Meier curves for disease-free survival (DFS) in patients with FIGO stage IVB endometrial cancer treated with surgery versus non-surgical management, after adjustment using inverse probability of treatment weighting (IPTW). Shaded areas represent 95% confidence intervals.

Similarly, IPTW-adjusted analyses for DFS showed significantly improved outcomes in the surgery group (Figure 5). The IPTW-weighted Cox model confirmed a significant reduction in the risk of recurrence or death associated with surgical treatment (HR 0.67, 95% CI 0.48–0.94, *p* = 0.02). These findings indicate that the survival benefit of surgery persisted after correction for baseline imbalances.

To provide causal estimates, subsequent analyses focused on the comparison between surgery versus no surgery rather than the original three treatment groups, allowing a clearer evaluation of the independent impact of surgical intervention.

### 3.3. Time-Period Subgroup Analysis

In the time-period subgroup analysis, the effect of surgery on survival varied across diagnostic eras (Supplementary Figure S1). The IPTW-weighted Cox regression results stratified by diagnostic period are shown in Table 4. In the contemporary cohort (2011–2019), surgical treatment was associated with a significantly reduced risk of death, whereas in the earlier period (2000–2010) the association was weaker and did not reach statistical significance. A similar pattern was observed for DFS, with a significant benefit of surgery in the later period but not in the earlier one. The corresponding IPTW-weighted DFS curves are shown in Supplementary Figure S2. These findings suggest that the survival advantage of surgery has become more pronounced over time, likely reflecting improvements in systemic therapy, optimized patient selection, and advances in perioperative management.

## 4. Discussion

This population-based registry study provides robust real-world evidence on treatment strategies and survival outcomes in patients with FIGO stage IVB endometrial cancer. Survival analysis demonstrated that combination therapy consisting of cytoreductive surgery plus adjuvant treatment achieved superior progression-free survival (PFS) and overall survival (OS) compared with surgery or non-surgical treatment alone. To the best of our knowledge, this represents the largest population-based investigation to date specifically assessing the role of cytoreductive surgery in patients with metastatic endometrial cancer.

To further enhance the robustness of our findings and minimize selection bias inherent to the non-random allocation of surgical treatment, we applied an inverse probability of treatment weighting (IPTW) approach based on propensity scores. This strategy generated a pseudo-population with well-balanced baseline characteristics, as confirmed by standardized mean differences and Love plots. Following IPTW adjustment, cytoreductive surgery remained independently associated with significantly improved OS and DFS. These results indicate that the observed survival benefit is unlikely to be solely attributable to preferential selection of younger or fitter patients for surgery, supporting the independent prognostic value of surgical cytoreduction in FIGO IVB disease. The consistency observed between crude analyses, conventional multivariable models, and IPTW-weighted analyses further strengthens the validity of our findings and underscores the relevance of cytoreductive surgery within real-world clinical practice.

A particularly relevant observation was the temporal variation in the prognostic effect of surgery. In the earlier diagnostic era (2000–2010), surgical treatment did not confer a statistically significant survival advantage, whereas this effect became evident and significant in the more recent period (2011–2019). This temporal shift likely reflects progress in systemic therapies, improvements in perioperative management, better imaging and staging accuracy, and more refined patient selection. Collectively, these factors suggest that the role of surgery has evolved and become more meaningful within modern multimodal treatment strategies.

Only a limited number of studies have previously demonstrated the benefits of multimodal approaches in advanced endometrial cancer. In a retrospective cohort of 58 patients, the combination of cytoreductive surgery and non-surgical therapy was associated with improved survival, with the most favorable outcomes observed in patients achieving no residual disease, followed by those with minimal residual tumor burden, and the poorest outcomes in patients who did not undergo cytoreduction [15]. This aligns well with our results. However, that study was restricted to patients with endometrioid histology, limiting its generalizability.

A substantial proportion of patients in our cohort underwent surgery without subsequent systemic therapy. This likely reflects historical treatment paradigms, postoperative complications, patient refusal, rapid disease progression, or contraindications to systemic therapy related to frailty or comorbidities. This observation highlights the heterogeneity of real-world management practices throughout the long study period and reinforces the need to interpret survival outcomes within the context of evolving therapeutic standards.

Kanno et al. demonstrated that macroscopic complete resection of intra-abdominal metastases significantly improved survival in patients with distant metastatic endometrial cancer, irrespective of whether surgery was performed before or after chemotherapy [10]. Our results are further supported by Bristow and colleagues, who demonstrated a survival advantage for cytoreductive surgery in patients with stage IV disease, including those with non-endometrioid histology [11]. In these studies, the most important determinant of outcome remained the volume of residual disease following surgery [16], reinforcing the critical role of maximal cytoreduction.

Consistent with prior literature, we observed that high tumor grade was significantly associated with poorer PFS and OS. Although non-endometrioid histology was not significantly different across treatment groups in this cohort, it remains well recognized as a marker of aggressive tumor biology and inferior outcomes. Notably, previous studies have shown that cytoreduction can still provide survival benefit in this subgroup [11], supporting the potential value of surgery even in biologically aggressive tumors.

A recent analysis comparing chemotherapy alone versus chemotherapy combined with hysterectomy in patients with uterine cancer and distant metastases reported a survival increase from 11 to 19.8 months when surgery was added [17]. However, that study included heterogeneous uterine malignancies, including sarcomas, thereby limiting direct comparability with our endometrial cancer-specific cohort. Interestingly, outcomes were more favorable when chemotherapy preceded surgical intervention, suggesting potential relevance of treatment sequencing.

Several limitations should be acknowledged. The retrospective nature of the study and the heterogeneity in treatment approaches introduce potential for bias. The exclusion of patients with missing FIGO staging or incomplete treatment data may also contribute to selection bias, although this was necessary to preserve data integrity. Additionally, combination therapy was more frequently administered to younger patients, reflecting potential treatment selection bias. While IPTW adjustment minimized this effect, residual confounding due to unmeasured variables cannot be entirely excluded.

Another important limitation is the grouping of heterogeneous non-surgical modalities into a single comparator category. Although chemotherapy and radiotherapy were commonly used, treatment strategies frequently overlapped and were unevenly distributed. Immunotherapy was administered in only 43 patients and predominantly in recent years. Stratification by specific modality would have resulted in small and clinically heterogeneous subgroups, compromising statistical power and interpretability. Therefore, grouping non-surgical therapies was necessary for analytical robustness. This limitation restricts direct comparability between individual non-surgical strategies and should be considered when interpreting the results, while the primary focus of the study remained the evaluation of the independent impact of surgery on survival outcomes.

The long recruitment period represents an additional limitation, given the rapid evolution of systemic treatments and the introduction of molecular classification. Many patients in earlier years were treated before modern targeted therapies and immune checkpoint inhibitors became standard, and treatment decisions were based primarily on clinicopathologic parameters. Consequently, results should be interpreted with caution when applying them to contemporary clinical settings. Nonetheless, this study offers valuable historical insight and establishes a benchmark for future evaluations in the era of molecular-guided therapy.

The strengths of this study include: (i) a large cohort with long-term follow-up; (ii) exclusive inclusion of FIGO IVB patients; (iii) direct comparison of treatment strategies; (iv) strong external validity due to the population-based design and minimal exclusion criteria; and (v) minimal loss to follow-up.

Ideally, the definitive evaluation of the role of cytoreductive surgery in advanced endometrial cancer should be accomplished through a randomized controlled trial comparing systemic therapy alone versus combined cytoreductive surgery and systemic treatment. However, such a design may pose ethical challenges. Therefore, further large-scale, high-quality observational studies with rigorous statistical methodologies remain essential to refine therapeutic strategies in this challenging clinical setting.

## 5. Conclusions

Overall, this study reinforces the central role of multimodal treatment strategies in improving survival outcomes for patients with FIGO stage IVB endometrial cancer. The significant survival benefit associated with the combination of cytoreductive surgery and adjuvant therapy supports the integration of surgical intervention into selected multimodal treatment pathways, in line with existing evidence.

However, given the heterogeneity of advanced-stage disease and evolving therapeutic standards, further high-quality studies are required to refine patient selection criteria and to more precisely identify those most likely to benefit from cytoreductive surgery. Future research should focus on integrating clinical, molecular, and treatment-related factors to optimize individualized therapeutic approaches in this challenging patient population.

## Figures and Tables

**Table 1:** Clinical and pathological characteristics.

Variables	OP	RT/CT/HT	OP + RT/CT/HT	*p*-Value
Age, median	71 (42–91)	72 (40–85)	65 (28–84)	<0.001
Histological type				0.458
Endometrioid	41 (50.6%)	36 (65.5%)	92 (58.2%)	
Non-endometrioid	37 (45.7%)	18 (32.7%)	59 (37.3%)	
Others	3 (3.7%)	1 (1.8%)	7 (4.4%)	
Grading				0.425
Low	27 (33.3%)	24 (44.4%)	58 (37.4%)	
High	54 (66.7%)	30 (55.6%)	97 (62.6%)	
Primary Metastases				
Bone	2 (2.5%)	12 (21.8%)	10 (6.3%)	<0.001
Lung	20 (24.7%)	28 (50.9%)	38 (24.1%)	<0.001
Liver	7 (8.6%)	7 (12.7%)	19 (12.0%)	0.681
Brain	3 (3.7%)	7 (12.7%)	12 (7.6%)	0.145
Abdominal	48 (59.3%)	15 (27.3%)	96(60.8%)	<0.001
Lymph nodes (distant)	10 (12.3%)	11 (20.0%)	17 (10.8%)	0.209
Other	9 (11.1%)	4(7.3%)	16 (10.1%)	0.752

**Table 2:** Uni- and multivariable analysis of PFS.

Variables	Univariable	*p*-Value	Multivariable	*p*-Value
	HR (95%CI)		HR (95%CI)	
RT/CT/HT vs. OP + RT/CT/HT	1 0.62 (0.44–0.88)	0.007	1 0.58 (0.39–0.86)	0.007
RT/CT/HT vs. OP	1 0.98 (0.67–1.45)	0.933	1 1.23 (0.77–1.97)	0.318
Age 69 years vs. >69 years	1 1.51 (1.17–1.96)	0.002	1 1.24 (0.89–1.72)	0.208
Low grading vs. High grading	1 1.80 (1.36–2.38)	<0.001	1 1.88 (1.32–2.68)	<0.001
Endometrioid vs. Non-endometrioid	1 1.51 (1.56–1.97)	0.003	1 1.25 (0.88–1.78)	0.216
One metastasis vs. >2 metastases	1 1.73 (1.32–2.28)	<0.001	1 1.50 (1.05–2.13)	0.024
None bone metastasis vs. Bone only metastasis	1 1.26 (0.79–2.02)	0.330	1 1.30 (0.75–2.26)	0.357
None visceral metastasis vs. Visceral metastasis	1 1.08 (0.75–1.54)	0.691	1 1.06 (0.67–1.67)	0.815

**Table 3:** Uni- and multivariable analysis of OS.

Variables	Univariable	*p*-Value	Multivariable	*p*-Value
	HR (95%CI)		HR (95%CI)	
RT/CT/HT vs. OP + RT/CT/HT	1 0.55 (0.39–0.78)	<0.001	1 0.49 (0.33–0.73)	<0.001
RT/CT/HT vs. OP	1 0.92 (0.62–1.36)	0.678	1 1.15 (0.71–1.85)	0.578
Age 69 years vs. >69 years	1 1.57 (1.21–2.04)	<0.001	1 1.26 (0.90–1.77)	0.173
Low grading vs. High grading	1 1.74 (1.31–2.31)	<0.001	1 1.83 (1.28–2.62)	<0.001
Endometrioid vs. Non-endometrioid	1 1.64 (1.25–2.15)	<0.001	1 1.47 (1.03–2.09)	0.034
One metastasis vs. >2 metastases	1 1.71 (1.30–2.26)	<0.001	1 1.38 (0.97–1.96)	0.070
None bone metastasis vs. Bone only metastasis	1 1.34 (0.83–2.17)	0.237	1 1.48 (0.84–2.61)	0.178
None visceral metastasis vs. Visceral metastasis	1 1.10 (0.76–1.60)	0.610	1 1.12 (0.69–1.82)	0.642

**Table 4:** IPTW-weighted Cox regression for surgery stratified by diagnostic period.

Outcome	Period	HR	95% Cl	*p*-Value
OS	2000–2010	0.70	0.42–1.18	0.18
OS	2011–2019	0.56	0.36–0.86	0.01
DFS	2000–2010	0.74	0.43–1.26	0.26
DFS	2011–2019	0.63	0.41–0.96	0.03

## Data Availability

The data presented in this study are not publicly available due to German data protection regulations and restrictions imposed by the regional cancer registry of Saxony-Anhalt. Access to the dataset is limited to authorized researchers and requires approval from the registry and the institutional ethics committee.

## Supplementary Materials

The following supporting information can be downloaded at: https://www.mdpi.com/article/10.3390/cancers17243965/s1, Figure S1: IPTW-weighted overall survival stratified by diagnostic period; Figure S2: IPTW-weighted disease-free survival stratified by diagnostic period.
