# Supplementary material for: Survival Impact of Cytoreductive Surgery in FIGO Stage IVB Endometrial Cancer: A Population-Based Study
Source: Cancers (Basel). 2025 Dec 12;17(24):3965. doi: 10.3390/cancers17243965 (PMC12730323; doi:10.3390/cancers17243965)
Supplement: Supplementary file 1 [file cancers-17-03965-s001.zip › cancers-3964058-supplementary.pdf]

A

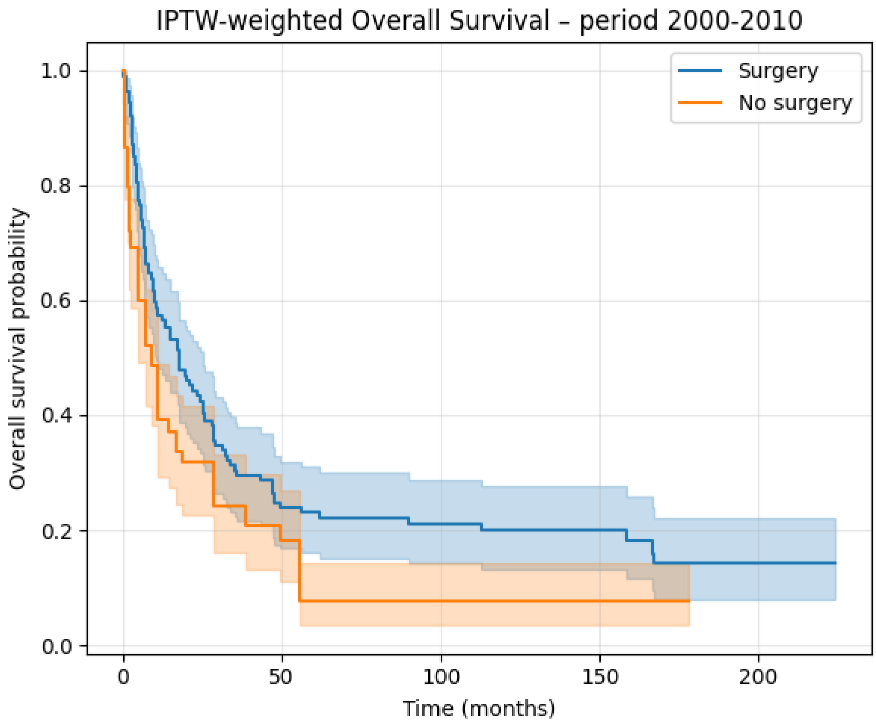

B

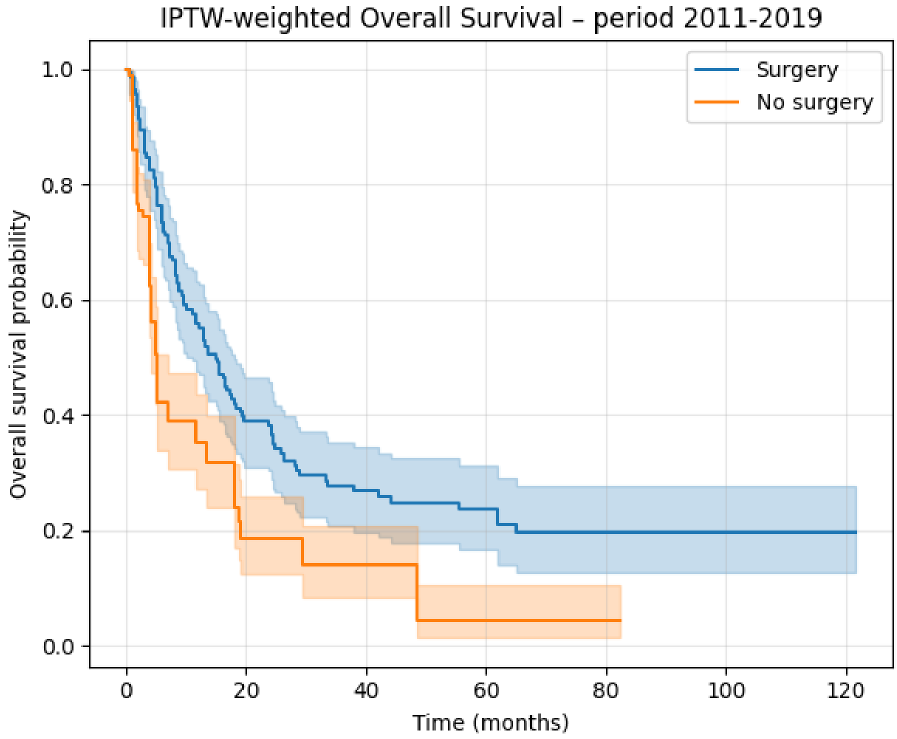

Figure S1. IPTW-weighted overall survival stratified by diagnostic period. A. IPTW-weighted Kaplan–Meier curves for OS stratified by diagnostic period (2000–2010). B. IPTW-weighted Kaplan–Meier curves for OS stratified by diagnostic period (2011–2019). IPTW-weighted Kaplan–Meier curves for overall survival stratified by diagnostic period (2000–2010 vs 2011–2019). Surgery was associated with a significantly improved survival in the contemporary period, whereas the effect was attenuated and not statistically significant in the earlier period. Shaded areas represent 95% confidence intervals.

A

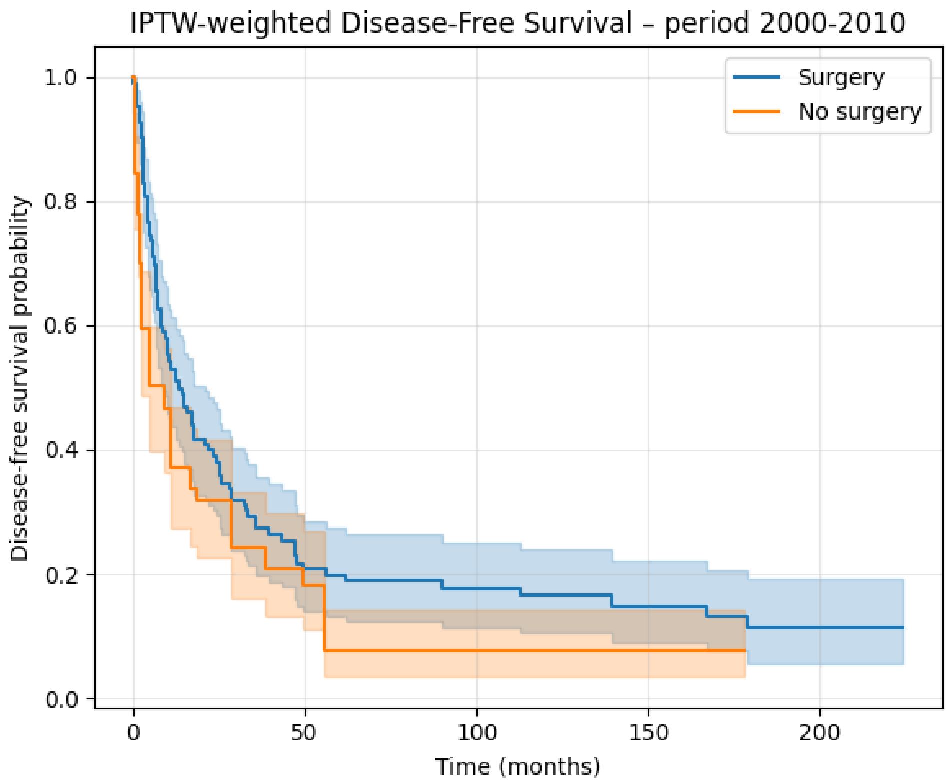

B

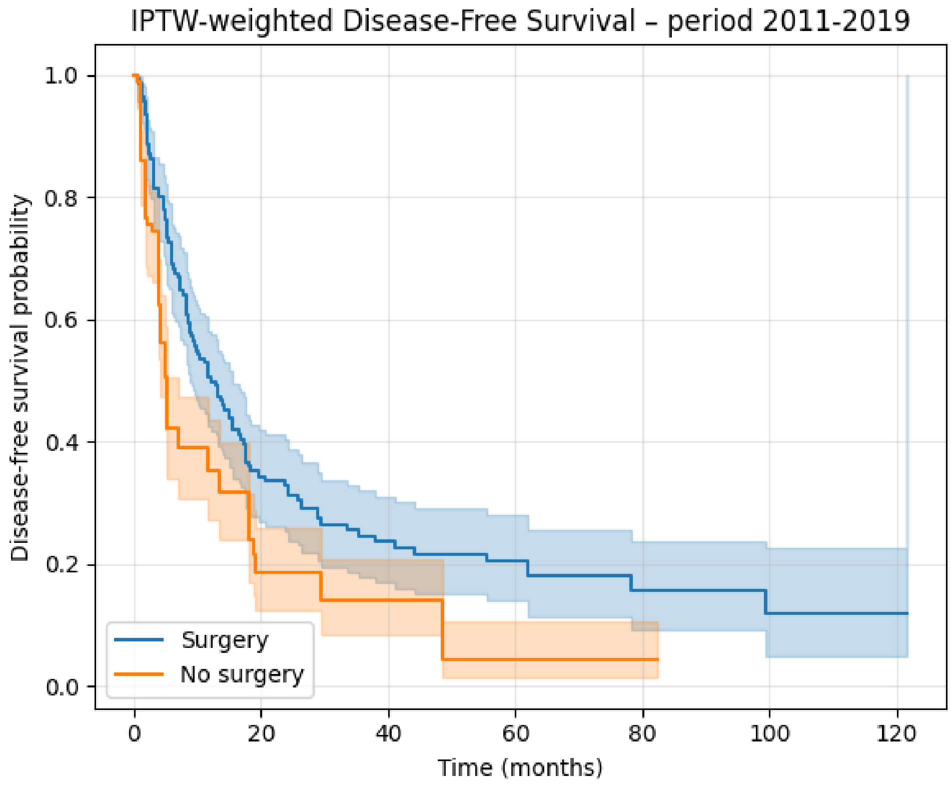

Figure S2. IPTW-weighted disease-free survival stratified by diagnostic period. A. IPTW-weighted Kaplan–Meier curves for DFS stratified by diagnostic period (2000–2010). B. IPTW-weighted Kaplan–Meier curves for DFS stratified by diagnostic period (2011–2019). IPTW-weighted Kaplan–Meier curves for disease-free survival stratified by diagnostic period (2000–2010 vs 2011–2019). The survival advantage associated with surgery was observed predominantly in the modern therapeutic era. Shaded areas represent 95% confidence intervals.
